# Tobacco Product Use Among Adults — United States, 2020

**DOI:** 10.15585/mmwr.mm7111a1

**Published:** 2022-03-18

**Authors:** Monica E. Cornelius, Caitlin G. Loretan, Teresa W. Wang, Ahmed Jamal, David M. Homa

**Affiliations:** 1 Office on Smoking and Health, National Center for Chronic Disease Prevention and Health Promotion, CDC.

Although cigarette smoking has declined over the past several decades, a diverse landscape of combustible and noncombustible tobacco products has emerged in the United States (*1*–*4*). To assess recent national estimates of commercial tobacco product use among U.S. adults aged ≥18 years, CDC analyzed data from the 2020 National Health Interview Survey (NHIS). In 2020, an estimated 47.1 million U.S. adults (19.0%) reported currently using any commercial tobacco product, including cigarettes (12.5%), e-cigarettes (3.7%), cigars (3.5%), smokeless tobacco (2.3%), and pipes* (1.1%).^†^ From 2019 to 2020, the prevalence of overall tobacco product use, combustible tobacco product use, cigarettes, e-cigarettes, and use of two or more tobacco products decreased. Among those who reported current tobacco product use, 79.6% reported using combustible products (e.g., cigarettes, cigars, or pipes), and 17.3% reported using two or more tobacco products.^§^ The prevalence of any current commercial tobacco product use was higher among the following groups: 1) men; 2) adults aged <65 years; 3) non-Hispanic American Indian or Alaska Native (AI/AN) adults and non-Hispanic adults categorized as of “Other” race^¶^; 4) adults in rural (nonmetropolitan) areas; 5) those whose highest level of educational attainment was a general educational development certificate (GED); 6) those with an annual household income <$35,000; 7) lesbian, gay, or bisexual adults; 8) uninsured adults or those with Medicaid; 9) adults living with a disability; and 10) those who regularly had feelings of anxiety or depression. Continued monitoring of tobacco product use and tailored strategies and policies that reduce the effects of inequitable conditions could aid in reducing disparities in tobacco use (*1*,*4*).

NHIS is an annual, nationally representative household survey of the noninstitutionalized U.S. civilian population.** In 2020, 31,568 adults aged ≥18 years (21,153 from the original 2020 sample [response rate: 48.9%] and 10,415 reinterviewed from 2019 [response rate: 29.6%]) participated^††^ (*5*). Data were weighted to provide nationally representative estimates, adjusting for differences in selection probability and nonresponse. As used in this report, “tobacco” refers to commercial tobacco products and not to tobacco used for medicinal and spiritual purposes by some American Indian communities. CDC assessed use of five tobacco products: cigarettes, cigars (cigars, cigarillos, or filtered little cigars), pipes (regular pipes, water pipes, or hookahs), e-cigarettes, and smokeless tobacco. Current cigarette smoking was defined as having ever smoked 100 or more cigarettes within one’s lifetime and smoking every day or some days at the time of survey. Current use of all other commercial tobacco products was defined as having reported use of these products every day or some days at the time of survey. Prevalence estimates for current use of any tobacco product, any combustible tobacco product, and two or more tobacco products were calculated. For 2020, estimates were calculated overall and by sex, age, race and ethnicity, U.S. Census region,^§§^ urban-rural designation,^¶¶^ education (for adults aged ≥25 years), marital status, annual household income,*** sexual orientation,^†††^ health insurance coverage,^§§§^ disability,^¶¶¶^ and regularly had feelings of anxiety or depression.****

CDC assessed statistically significant (p<0.05) differences in current cigarette smoking by urban-rural designation among each racial and ethnic group, changes in prevalence of tobacco product use during 2019 and 2020, and changes in average number of cigarettes smoked per day (1–9, 10–19, 20–29, and ≥30 cigarettes) during 2005–2020. SAS-callable SUDAAN software (version 11.0.3; Research Triangle Institute) was used to conduct all analyses. This activity was reviewed by CDC and was conducted consistent with applicable federal law and CDC policy.^††††^

Among U.S. adults in 2020, 19.0% (estimated 47.1 million) currently used any tobacco product, 15.2% (37.5 million) used any combustible tobacco product, and 3.3% (8.1 million) used two or more tobacco products. Cigarettes were the most commonly used tobacco product (12.5%; 30.8 million). Prevalence of use and estimated number of users of other tobacco products in 2020 was as follows: e-cigarettes (3.7%; 9.1 million), cigars (3.5%; 8.6 million), smokeless tobacco (2.3%; 5.7 million), and pipes (1.1%; 2.6 million) (Table). Among persons who currently used any tobacco product, 79.6% used combustible tobacco products, and 17.3% reported using two or more tobacco products. From 2019 to 2020, statistically significant decreases (p<0.05) were observed in the prevalence of use of any tobacco product (20.8% to 19.0%; p<0.001), combustible tobacco products (16.7% to 15.2%; p<0.001), two or more tobacco products (3.9% to 3.3%; p = 0.003), cigarettes (14.0% to 12.5%; p<0.001), and e-cigarettes (4.5% to 3.7%; p<0.001). No statistically significant changes in past-year prevalence were observed among other products, including cigars (3.6% to 3.5%; p = 0.60) and pipes (1.0% to 1.1%; p = 0.44), and smokeless products (2.4% to 2.3%; p = 0.50).

**TABLE:** Percentage of adults aged ≥18 years who reported tobacco product use “every day” or “some days,” by tobacco product and selected characteristics — National Health Interview Survey, United States, 2020

Characteristic	Tobacco product use,* % (95% CI)^†^							
	Any tobacco product^§^	Combustible tobacco product^¶^	Cigarettes**	Cigars^††^	Pipes^§§^	E-cigarettes^¶¶^	Smokeless tobacco products***	Two or more tobacco products^†††^
**Overall**	**19.0 (18.4–19.7)**	**15.2 (14.6–15.8)**	**12.5 (11.9–13.0)**	**3.5 (3.2–3.8)**	**1.1 (0.9–1.3)**	**3.7 (3.4–4.0)**	**2.3 (2.1–2.6)**	**3.3 (3.0–3.6)**
**Sex**								
Men	24.5 (23.5–25.5)	18.8 (17.9–19.8)	14.1 (13.3–14.9)	6.3 (5.8–6.9)	1.5 (1.2–1.8)	4.6 (4.2–5.2)	4.5 (4.0–5.0)	5.2 (4.7–5.8)
Women	13.9 (13.2–14.7)	11.7 (11.1–12.4)	11.0 (10.3–11.6)	0.8 (0.7–1.0)	0.7 (0.5–0.9)	2.8 (2.5–3.2)	0.3 (0.2–0.5)	1.5 (1.2–1.8)
**Age group, yrs**								
18–24	17.6 (15.5–19.9)	10.9 (9.2–12.9)	7.4 (5.9–9.0)	4.1 (3.1–5.4)	2.1 (1.3–3.1)	9.4 (7.8–11.2)	2.4 (1.6–3.4)	5.7 (4.4–7.2)
25–44	22.9 (21.8–24.0)	18.0 (16.9–19.1)	14.1 (13.1–15.1)	5.0 (4.4–5.6)	1.7 (1.3–2.1)	5.2 (4.6–5.7)	2.8 (2.4–3.3)	4.9 (4.3–5.6)
45–64	20.4 (19.4–21.5)	16.9 (16.0–17.9)	14.9 (14.0–15.9)	2.8 (2.5–3.2)	0.6 (0.4–0.8)	2.2 (1.9–2.6)	2.5 (2.1–3.0)	2.3 (1.9–2.6)
≥65	11.8 (10.9–12.7)	10.4 (9.6–11.3)	9.0 (8.2–9.8)	1.8 (1.5–2.1)	0.3 (0.2–0.5)	0.6 (0.4–0.8)	1.2 (0.9–1.6)	1.0 (0.8–1.3)
**Race and ethnicity** ^§§§^								
American Indian or Alaska Native, non-Hispanic	34.9 (24.8–46.2)	29.3 (18.8–41.7)	27.1 (17.4–38.6)	—^¶¶¶^	—^¶¶¶^	—^¶¶¶^	6.8 (3.6–11.5)	10.9 (6.4–16.9)
White, non-Hispanic	21.1 (20.4–21.9)	16.3 (15.6–17.0)	13.3 (12.7–14.0)	3.8 (3.4–4.2)	0.9 (0.8–1.2)	4.2 (3.8–4.7)	3.2 (2.8–3.5)	3.6 (3.2–3.9)
Black, non-Hispanic	19.4 (17.4–21.5)	18.0 (16.2–19.9)	14.4 (12.6–16.3)	4.6 (3.7–5.6)	1.6 (1.1–2.3)	1.6 (1.0–2.3)	0.8 (0.4–1.5)	2.9 (2.2–3.9)
Asian, non-Hispanic	11.5 (9.6–13.7)	8.7 (7.0–10.7)	8.0 (6.4–9.9)	0.9 (0.4–1.6)	0.4 (0.1–0.9)	3.4 (2.3–4.7)	0.4 (0.2–0.9)	1.4 (0.8–2.3)
Other, non-Hispanic	29.1 (24.1–34.4)	21.0 (16.3–26.4)	19.5 (14.9–24.7)	—^¶¶¶^	—^¶¶¶^	7.8 (5.1–11.2)	3.7 (1.9–6.4)	9.2 (5.3–14.8)
Hispanic	11.7 (10.4–13.1)	9.8 (8.6–11.0)	8.0 (7.0–9.2)	2.2 (1.7–2.8)	0.9 (0.6–1.4)	2.8 (2.2–3.5)	0.4 (0.2–0.7)	2.2 (1.7–2.8)
U.S. Census region****								
Northeast	16.6 (15.0–18.3)	13.4 (12.1–14.8)	10.4 (9.3–11.5)	3.1 (2.5–3.8)	0.8 (0.5–1.2)	3.0 (2.4–3.8)	1.6 (1.1–2.3)	2.1 (1.7–2.6)
Midwest	22.0 (20.6–23.4)	18.2 (16.8–19.6)	15.2 (14.0–16.5)	3.7 (3.1–4.4)	1.3 (0.9–1.8)	4.1 (3.4–4.8)	2.6 (2.1–3.2)	4.1 (3.3–4.9)
South	21.1 (20.0–22.2)	16.9 (15.8–18.0)	14.1 (13.1–15.2)	4.1 (3.6–4.7)	1.1 (0.8–1.4)	3.6 (3.2–4.2)	2.7 (2.3–3.2)	3.7 (3.2–4.3)
West	15.0 (13.9–16.1)	11.1 (10.2–12.0)	9.0 (8.2–9.8)	2.5 (2.1–3.0)	1.1 (0.8–1.5)	4.0 (3.3–4.7)	1.9 (1.5–2.4)	2.8 (2.3–3.3)
**Metropolitan statistical area** ^††††^								
Urban	17.7 (17.0–18.4)	14.2 (13.5–14.8)	11.4 (10.8–12.0)	3.4 (3.1–3.8)	1.1 (0.9–1.3)	3.7 (3.3–4.0)	1.7 (1.5–2.0)	3.0 (2.7–3.3)
Rural	27.3 (25.5–29.2)	21.3 (19.6–23.1)	19.0 (17.4–20.8)	3.7 (2.9–4.7)	1.0 (0.6–1.5)	3.9 (3.0–5.0)	5.9 (4.8–7.0)	5.0 (4.0–6.1)
**Education (adults aged ≥25 yrs)**								
0–12 yrs (no diploma)	24.8 (22.3–27.4)	22.7 (20.3–25.2)	21.5 (19.2–24.0)	3.1 (2.2–4.2)	0.7 (0.3–1.3)	1.4 (0.8–2.2)	2.4 (1.7–3.4)	3.6 (2.6–4.9)
GED	40.5 (35.4–45.8)	34.5 (29.5–39.7)	32.0 (27.2–37.2)	5.9 (3.9–8.5)	1.6 (0.5–3.6)	5.4 (3.5–7.9)	3.8 (2.1–6.2)	6.8 (4.7–9.5)
High school diploma	24.2 (22.9–25.6)	19.6 (18.3–20.9)	17.6 (16.4–18.9)	3.1 (2.5–3.6)	0.9 (0.6–1.4)	3.5 (3.0–4.2)	3.3 (2.7–4.0)	3.8 (3.1–4.5)
Some college, no diploma	21.7 (20.2–23.3)	17.3 (15.9–18.7)	14.4 (13.1–15.7)	4.0 (3.3–4.8)	0.8 (0.5–1.2)	4.1 (3.4–5.0)	2.6 (2.0–3.3)	3.5 (2.8–4.3)
Associate degree (academic or technical/vocational)	19.4 (17.8–21.1)	15.3 (13.8–16.8)	12.7 (11.3–14.1)	3.6 (2.8–4.5)	1.0 (0.6–1.6)	3.7 (2.9–4.5)	2.6 (2.0–3.4)	3.3 (2.5–4.2)
Bachelor’s degree	11.7 (10.7–12.6)	9.0 (8.2–9.9)	5.6 (5.0–6.3)	3.3 (2.8–3.9)	1.0 (0.7–1.4)	2.4 (2.0–2.9)	1.3 (1.0–1.7)	1.7 (1.4–2.1)
Graduate degree (master's, professional, or doctoral)	8.6 (7.6–9.7)	6.9 (6.0–7.9)	3.5 (2.9–4.1)	3.0 (2.5–3.7)	0.9 (0.5–1.4)	1.5 (1.1–2.1)	0.8 (0.6–1.2)	1.1 (0.8–1.6)
**Marital status**								
Married/Living with partner	17.5 (16.7–18.2)	13.8 (13.1–14.5)	10.9 (10.3–11.6)	3.6 (3.2–4.0)	0.8 (0.6–1.0)	3.1 (2.7–3.4)	2.6 (2.3–2.9)	2.9 (2.5–3.3)
Divorced/Separated/Widowed	21.6 (20.3–22.9)	18.9 (17.6–20.1)	17.3 (16.1–18.5)	2.3 (1.8–2.8)	0.8 (0.5–1.1)	2.6 (2.1–3.1)	1.6 (1.2–2.1)	2.6 (2.1–3.2)
Single/Never married/Not living with a partner	21.4 (20.0–23.0)	16.3 (14.9–17.7)	13.0 (11.7–14.4)	4.0 (3.4–4.8)	2.1 (1.6–2.7)	6.2 (5.3–7.1)	2.2 (1.7–2.9)	4.8 (4.1–5.7)
**Annual household income, $** ^§§§§^								
<35,000	25.2 (23.8–26.5)	22.1 (20.9–23.4)	20.2 (19.0–21.4)	3.0 (2.6–3.5)	1.5 (1.1–2.0)	3.7 (3.1–4.3)	1.9 (1.4–2.4)	4.1 (3.6–4.8)
35,000–74,999	20.3 (19.2–21.5)	16.4 (15.3–17.5)	14.1 (13.1–15.1)	3.6 (3.0–4.1)	0.9 (0.6–1.2)	3.9 (3.3–4.5)	2.3 (2.0–2.8)	3.6 (3.1–4.2)
75,000–99,999	18.4 (16.8–20.1)	13.2 (11.8–14.7)	10.5 (9.3–11.9)	3.3 (2.5–4.1)	1.0 (0.5–1.5)	4.5 (3.6–5.6)	3.1 (2.4–4.0)	3.4 (2.6–4.4)
≥100,000	13.7 (12.8–14.7)	9.9 (9.1–10.7)	6.2 (5.6–6.9)	3.8 (3.4–4.3)	1.0 (0.7–1.4)	3.2 (2.7–3.7)	2.3 (1.9–2.7)	2.3 (1.9–2.8)
**Sexual orientation**								
Heterosexual/Straight	18.8 (18.2–19.5)	15.0 (14.4–15.6)	12.3 (11.7–12.8)	3.5 (3.2–3.8)	1.0 (0.9–1.2)	3.5 (3.2–3.8)	2.4 (2.2–2.7)	3.2 (2.9–3.5)
Lesbian, gay, or bisexual	25.1 (21.4–29.1)	18.9 (15.3–22.8)	16.1 (12.7–19.9)	4.3 (2.4–7.1)	2.6 (1.2–4.9)	8.7 (6.5–11.4)	0.8 (0.3–1.6)	6.2 (3.9–9.4)
**Health insurance coverage** ^¶¶¶¶^								
Private insurance	16.4 (15.7–17.2)	12.3 (11.7–12.9)	9.2 (8.6–9.7)	3.5 (3.2–3.9)	0.9 (0.8–1.2)	3.8 (3.4–4.2)	2.4 (2.1–2.7)	2.8 (2.5–3.1)
Medicaid	28.6 (26.5–30.8)	24.6 (22.6–26.6)	22.7 (20.8–24.8)	3.0 (2.3–3.8)	1.9 (1.3–2.8)	4.4 (3.4–5.6)	2.4 (1.7–3.3)	5.0 (3.9–6.2)
Medicare only (aged ≥65 yrs)	12.5 (11.0–14.2)	11.3 (9.8–12.9)	10.2 (8.7–11.8)	1.6 (1.1–2.2)	0.1 (0.0–0.3)	0.7 (0.4–1.0)	1.1 (0.7–1.6)	1.0 (0.6–1.6)
Other public insurance	21.3 (18.9–24.0)	17.7 (15.3–20.3)	14.8 (12.6–17.4)	4.2 (3.1–5.6)	1.0 (0.5–1.7)	2.7 (1.9–3.8)	2.4 (1.6–3.4)	3.1 (2.1–4.2)
Uninsured	27.3 (25.0–29.8)	23.3 (21.1–25.6)	21.2 (19.1–23.4)	4.8 (3.7–6.1)	1.6 (1.0–2.3)	5.1 (4.0–6.4)	2.5 (1.8–3.4)	6.0 (4.8–7.4)
**Disability** *****								
Yes	25.4 (23.3–27.6)	21.6 (19.6–23.8)	19.8 (17.8–22.0)	3.4 (2.5–4.6)	1.2 (0.8–1.7)	3.5 (2.7–4.5)	2.9 (2.1–4.1)	4.8 (3.6–6.1)
No	18.4 (17.8–19.1)	14.6 (14.0–15.2)	11.8 (11.2–12.3)	3.5 (3.2–3.8)	1.1 (0.9–1.3)	3.7 (3.4–4.1)	2.3 (2.0–2.5)	3.2 (2.8–3.5)
**Regularly having feelings of anxiety** ^†††††^								
Yes	29.6 (27.7–31.5)	24.1 (22.2–26.0)	21.4 (19.6–23.2)	4.1 (3.3–5.0)	1.8 (1.3–2.6)	7.1 (5.9–8.4)	2.1 (1.5–2.9)	5.6 (4.6–6.6)
No	17.7 (17.0–18.4)	14.0 (13.4–14.7)	11.3 (10.8–11.9)	3.4 (3.1–3.7)	1.0 (0.8–1.2)	3.3 (3.0–3.6)	2.3 (2.1–2.6)	3.0 (2.7–3.3)
**Regularly having feelings of depression** ^§§§§§^								
Yes	35.6 (32.4–39.0)	29.6 (26.6–32.8)	26.9 (23.9–30.0)	3.7 (2.6–5.1)	2.8 (1.6–4.6)	8.3 (6.4–10.6)	2.6 (1.5–4.3)	6.7 (4.9–9.0)
No	18.3 (17.6–18.9)	14.5 (13.9–15.1)	11.8 (11.2–12.3)	3.5 (3.2–3.8)	1.0 (0.8–1.2)	3.5 (3.2–3.8)	2.3 (2.1–2.6)	3.1 (2.8–3.4)

**Abbreviation:** GED = general educational development certificate.

* Smoking and tobacco use here refer to use of commercial tobacco products and not to tobacco used for medicinal and spiritual purposes by some American Indian communities.

^†^ 95% Korn-Graubard CIs. National Center for Health Statistics data presentation standards. https://www.cdc.gov/nchs/data/series/sr_02/sr02_175.pdf

^§^ Any tobacco use was defined as use either “every day” or “some days” of at least one tobacco product. (For cigarettes, users were defined as adults who reported use either “every day” or “some days” and had smoked 100 or more cigarettes during their lifetime).

^¶^ Any combustible tobacco use was defined as use either “every day” or “some days” of at least one combustible tobacco product: cigarettes; cigars, cigarillos, filtered little cigars; pipes, water pipes, or hookah. (For cigarettes, users were defined as adults who reported use either “every day” or “some days” and had smoked 100 or more times during their lifetime).

** Current cigarette smoking was defined as smoking 100 or more cigarettes during a person’s lifetime and now smoking cigarettes “every day” or “some days.”

^††^ Current cigar smoking was defined as smoking cigars, cigarillos, or little filtered cigars at least once during a person’s lifetime and now smoking at least one of these products “every day” or “some days.”

^§§^ Current pipe smoking was defined as smoking tobacco in a regular pipe, water pipe, or hookah at least once during a person’s lifetime and now smoking at least one of these products “every day” or “some days.”

^¶¶^ Current e-cigarette use was defined as using e-cigarettes at least once during a person’s lifetime and now using e-cigarettes “every day” or “some days.”

*** Current smokeless tobacco product use was defined as using chewing tobacco, snuff, dip, snus, or dissolvable tobacco at least once during a person’s lifetime and now using at least one of these products “every day” or “some days.”

^†††^ Current multiple tobacco product use was defined as use “every day” or “some days” for at least two or more of the following tobacco products: cigarettes (100 or more cigarettes during lifetime); cigars, cigarillos, filtered little cigars; pipes, water pipes, or hookahs; e-cigarettes; or smokeless tobacco products.

^§§§^ Hispanic persons could be of any race. All other groups were non-Hispanic. The following four non-Hispanic single-race categories were available for sample adults in the 2020 National Health Interview Survey public use files: 1) White; 2) Black or African American; 3) Asian, and 4) American Indian or Alaska Native. Exclusive from these groups, the “Other, non-Hispanic” category includes those adults who were categorized as “non-Hispanic American Indian or Alaska Native and any other group” or “other single and multiple races.”

^¶¶¶^ Based on National Center for Health Statistics data presentation standards, estimates were statistically unreliable (https://www.cdc.gov/nchs/data/series/sr_02/sr02_175.pdf). SAS MACRO used for suppression criteria check. https://www.sas.com/content/dam/SAS/support/en/sas-global-forum-proceedings/2019/3659-2019.pdf

**** *Northeast:* Connecticut, Maine, Massachusetts, New Hampshire, New Jersey, New York, Pennsylvania, Rhode Island, and Vermont; *Midwest:* Illinois, Indiana, Iowa, Kansas, Michigan, Minnesota, Missouri, Nebraska, North Dakota, Ohio, South Dakota, and Wisconsin; *South:* Alabama, Arkansas, Delaware, District of Columbia, Florida, Georgia, Kentucky, Louisiana, Maryland, Mississippi, North Carolina, Oklahoma, South Carolina, Tennessee, Texas, Virginia, and West Virginia; *West:* Alaska, Arizona, California, Colorado, Hawaii, Idaho, Montana, Nevada, New Mexico, Oregon, Utah, Washington, and Wyoming.

^††††^ Urban = large central metropolitan, large fringe metropolitan, medium metropolitan, and small metropolitan; rural = nonmetropolitan. Metropolitan statistical areas are based on the 2013 National Center for Health Statistics Urban-Rural Classification Scheme for Counties. https://www.cdc.gov/nchs/data/series/sr_02/sr02_166.pdf

^§§§§ ^Based on the imputed sample adult family income (grouped) variable.

^¶¶¶¶^ Private insurance coverage includes adults who had any comprehensive private insurance plan (including health maintenance organizations and preferred provider organizations). Medicaid for adults aged <65 years includes adults who do not have private coverage, but who have Medicaid or other state-sponsored health plans including Children’s Health Insurance Program; for adults aged ≥65 years, includes adults aged ≥65 years who do not have any private coverage but have Medicare and Medicaid or other state-sponsored health plans. Medicare coverage only includes adults aged ≥65 years who only have Medicare coverage. Other public insurance includes adults who do not have private insurance, Medicaid, or other public coverage but have any type of military coverage, coverage from other government programs, or Medicare (adults aged <65 years). Uninsured includes adults who have not indicated that they are covered under private health insurance, Medicare, Medicaid, a state-sponsored health plan, other government programs, or military coverage. Insurance coverage is as of time of survey.

***** Disability was defined based on self-reported presence of selected limitations including vision, hearing, mobility, remembering or concentrating, self-care, and communication. Respondents had to answer, “A lot of difficulty” or “Cannot do at all/unable to do” to one of the following questions: “Do you have difficulty seeing, even when wearing glasses?,” “Do you have difficulty hearing, even when using a hearing aid?,” “Do you have any difficulty walking or climbing steps?,” “Using your usual language, do you have difficulty communicating, for example, understanding or being understood?,” “Do you have difficulty remembering or concentrating?,” “Do you have difficulty with self-care, such as washing all over or dressing?” to be coded as living with a disability; those who responded “no difficulty” or “some difficulty” to all six questions were coded as having no disability. Classifications are based on the 2020 National Health Interview Survey Washington Group Short Set Composite Disability Indicator recode, as based on the short set of questions recommended by the Washington Group on Disability Statistics. https://www.cdc.gov/nchs/washington_group/index.htm

^†††††^ Regularly having feelings of anxiety was assessed by the questions, “How often do you feel worried, nervous or anxious? Would you say daily, weekly, monthly, a few times a year, or never?” and “Thinking about the last time you felt worried, nervous or anxious, how would you describe the level of these feelings? Would you say a little, a lot, or somewhere in between?” Respondents indicating 1) feeling worried, nervous, or anxious daily and describing the level of those feelings as “somewhere in between a little and a lot” or “a lot” or 2) feeling worried, nervous, or anxious weekly and describing the level of those feelings as “a lot” were considered as regularly having feelings of anxiety. Those who answered 1) “never” feeling worried, nervous or anxious and who did not answer the question on the level of the feelings, 2) feeling worried, nervous or anxious and daily and described the level of those feelings as “a little,” 3) feeling worried, nervous, or anxious weekly and described the level of those feelings as “a little” or “somewhere in between a little and a lot,” or 4) feeling worried, nervous, or anxious “monthly” or “a few times a year” and described the level of those feelings as “a little”, “a lot” or “somewhere in between a little and a lot” were considered as not regularly having feelings of anxiety. Others not falling within those combinations were excluded. More information on the definition is available at https://wwwn.cdc.gov/NHISDataQueryTool/ER_Quarterly/index_quarterly.html, and more information on the question source is available at https://www.washingtongroup-disability.com/fileadmin/uploads/wg/Documents/Questions/WG_Implementation_Document__4C_-_WG-SS_Enhanced_Question_Specifications.pdf.

^§§§§§^ Regularly having feelings of depression was assessed by the questions, “How often do you feel depressed? Would you say daily, weekly, monthly, a few times a year, or never?” and “Thinking about the last time you felt depressed, how would you describe the level of these feelings? Would you say a little, a lot, or somewhere in between?” Respondents indicating 1) feeling depressed daily and describing the level of those feelings as “somewhere in between a little and a lot” or “a lot” or 2) feeling depressed weekly and describing the level of those feelings as “a lot” were considered as regularly having feelings of depression. Those who answered 1) “never” feeling depressed and who did not answer the question on the level of the feelings, 2) feeling depressed daily and described the level of those feelings as “a little,” 3) feeling depressed weekly and described the level of those feelings as “a little” or “somewhere in between a little and a lot,” or 4) feeling depressed “monthly” or “a few times a year” and described the level of those feelings as “a little,” “a lot” or “somewhere in between a little and a lot” were considered as not having feelings of depression. Others not falling within those combinations were excluded. More information on the definition is available at https://wwwn.cdc.gov/NHISDataQueryTool/ER_Quarterly/index_quarterly.html, and more information on the question source is available at https://www.washingtongroup-disability.com/fileadmin/uploads/wg/Documents/Washington_Group_Questionnaire__3_-_WG_Short_Set_on_Functioning_-_Enhanced.pdf.

Current cigarette smoking prevalence was higher among persons who resided in rural areas than among those who resided in urban areas among non-Hispanic Black (38% higher), Hispanic (38% higher) and non-Hispanic White (62% higher) adults; in contrast, prevalence among non-Hispanic Asian adults was 32% higher among those in urban areas (p<0.05) (Figure 1). Among adults who smoked cigarettes daily, the percentage who reported smoking 20–29 cigarettes per day decreased from 34.9% in 2005 to 27.9% in 2020, and the percentage who reported smoking 30 or more cigarettes per day decreased from 12.7% to 6.4%; the percentage who reported smoking 1–9 cigarettes per day increased from 16.4% to 25.0%, and the percentage who reported smoking 10–19 cigarettes per day increased from 36.0% to 40.7% (all p<0.001) (Figure 2).

**FIGURE 1:**
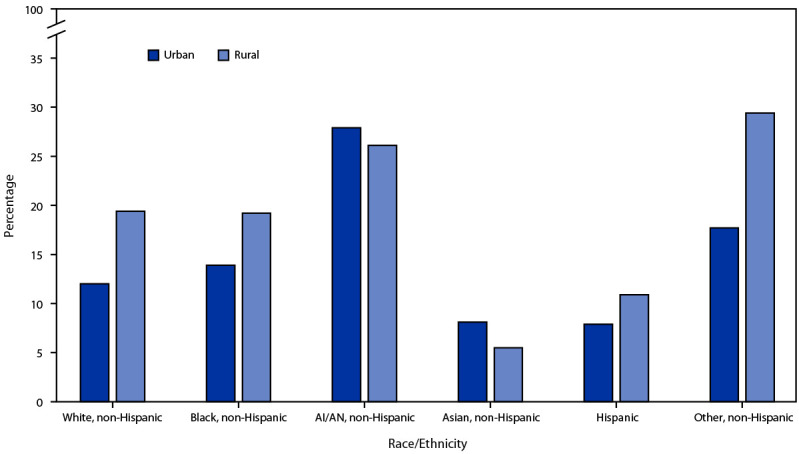
Prevalence of current cigarette smoking* among U.S. adults, by urban-rural^†^ designation and race and ethnicity^§^ — United States, 2020^¶^ **Abbreviation:** AI/AN = American Indian or Alaska Native. * Smoking and tobacco use here refer to use of commercial tobacco products and not to tobacco used for medicinal and spiritual purposes by some American Indian communities. ^†^ Urban = large central metropolitan, large fringe metropolitan, medium metropolitan, and small metropolitan; rural = nonmetropolitan. https://www.cdc.gov/nchs/data/series/sr_02/sr02_166.pdf ^§^ Hispanic adults could be of any race. All other groups were non-Hispanic. The following four non-Hispanic single-race categories were available for sample adults in the 2020 National Health Interview Survey public use files: 1) White, 2) Black or African American, 3) Asian, and 4) AI/AN. Exclusive from these groups, the “non-Hispanic, Other” category in this report includes those adults who were categorized as “non-Hispanic AI/AN and any other group” or “other single and multiple races.” The only multiracial categories available were “non-Hispanic AI/AN and any other group” and “other single and multiple races.” https://ftp.cdc.gov/pub/Health_Statistics/NCHS/Dataset_Documentation/NHIS/2020/srvydesc-508.pdf ^¶^ p<0.05 for differences in urban-rural cigarette smoking prevalence for the following race/ethnicity groups: non-Hispanic Asian, non-Hispanic Black, non-Hispanic White, Hispanic.

**FIGURE 2:**
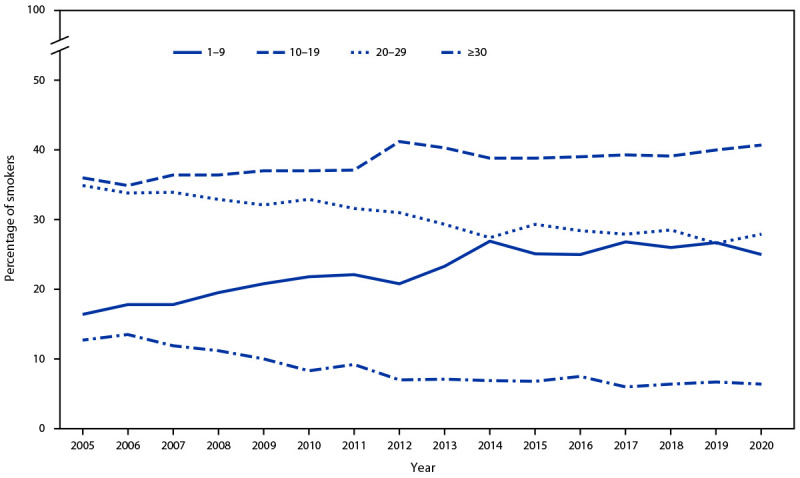
Percentage of adults aged ≥18 years who reported smoking cigarettes* every day, by average number of cigarettes smoked per day — United States, 2005–2020^†§^ * Smoking and tobacco use here refer to use of commercial tobacco products and not to tobacco used for medicinal and spiritual purposes by some American Indian communities. ^†^ Linear trends were adjusted for sex, age, race, and ethnicity. During 2005–2020, prevalence of adults who smoked daily and smoked 1–9 cigarettes per day and 10–19 cigarettes per day significantly increased (p<0.05); prevalence of adults who smoked daily and smoked 20–29 cigarettes per day and ≥30 cigarettes per day significantly deceased (p<0.05). ^§^ Changes in weighting and design methodology for the 2019 National Health Interview Survey could affect comparisons of weighted survey estimates over time; preliminary evaluation showed that the estimate of current cigarette smoking was affected by methodological changes, which might have shifted the estimate upward by 0.5 percentage points. In addition, changes in the 2020 National Health Interview Survey administration from in-person to primarily telephone-based might affect estimates. Under- and overrepresentation of certain groups exists. How this might bias the measured prevalence of current cigarette smoking is uncertain. For these reasons, observed trends should be interpreted cautiously. https://ftp.cdc.gov/pub/Health_Statistics/NCHS/Dataset_Documentation/NHIS/2019/srvydesc-508.pdf and https://www.cdc.gov/nchs/data/nhis/earlyrelease/EarlyRelease202009-508.pdf

The prevalence of any current tobacco product use was higher among 1) men (24.5%) than among women (13.9%); 2) persons aged 25–44 years (22.9%), 45–64 years (20.4%), or 18–24 years (17.6%) than among those aged ≥65 years (11.8%); 3) non-Hispanic AI/AN (34.9%), non-Hispanic Other (29.1%), non-Hispanic White (21.1%), and non-Hispanic Black (19.4%) adults than among Hispanic (11.7%) and non-Hispanic Asian (11.5%) adults; 4) persons living in the Midwest (22.0%) or the South (21.1%) than among those living in the Northeast (16.6%) or West (15.0%); 5) persons from rural areas (27.3%) than among those from urban areas (17.7%); and 6) persons with a GED (40.5%) than among those with other levels of education (Table). The prevalence of any current tobacco product use was also higher among 1) persons who were divorced/separated/widowed (21.6%) or single/never married/not living with a partner (21.4%) than among those married/living with a partner (17.5%); 2) persons who had an annual household income of <$35,000 (25.2%) than those with higher income; 3) lesbian, gay, or bisexual adults (25.1%) than heterosexual/straight adults (18.8%); 4) persons insured by Medicaid (28.6%) or who were uninsured (27.3%), than those who had some other public insurance (21.3%), private insurance (16.4%) or Medicare only (12.5%); 5) persons with a disability (25.4%) than those who did not (18.4%); and 6) persons who reported regularly having feelings of anxiety (29.6%) or depression (35.6%) than those who did not.

## Discussion

From 2019 to 2020 the prevalence of any commercial tobacco product use and use of certain commercial tobacco products decreased, yet nearly one in five adults (47.1 million) continued to use commercial tobacco products. Approximately three quarters of adults who used tobacco products used combustible products, with 30.8 million adults currently smoking cigarettes. Among all tobacco products, cigarettes and other combustible tobacco products are the predominant cause of tobacco-related morbidity and mortality (*1*). Increasing the use of evidence-based commercial tobacco control interventions (e.g., raising the price of tobacco products, smoke-free policies in public places, and increasing equitable cessation access) can help prevent tobacco product use initiation and increase cessation, further reducing tobacco use prevalence and related disease (*1*,*6*,*7*).

From 2005 to 2020, shifts were seen in cigarette use patterns among adults who smoked daily, with adults generally smoking fewer cigarettes per day in 2020 than in 2005. In 2020, 12.5% of U.S. adults aged ≥18 years smoked cigarettes, the lowest prevalence since data became available starting in 1965 (*1*). Factors that might have contributed to the lower prevalence of tobacco product use include high-impact antitobacco media campaigns (e.g., Tips from Former Smokers and Every Try Counts) and policies (e.g., smoke-free policies in public places and limiting the availability of specific types of tobacco products such as flavored products) at the local, tribal, state, and national level (*1*,*4*,*7*,*8*).

In 2020, marked sociodemographic differences in smoking prevalence among U.S. adults were observed, as well as differences between adults of different races and ethnicities by urban-rural designation. Among non-Hispanic Black, Hispanic, and non-Hispanic White adults, prevalence of cigarette smoking was higher among persons who resided in rural areas than their racial and ethnic counterparts in urban areas. The tobacco industry has historically targeted rural and low-income areas with increased advertising, price promotions, and access to tobacco retailers, thereby contributing to an environment where tobacco use is viewed as normal (*8*). Targeted marketing of menthol cigarettes to non-Hispanic Black and Hispanic racial and ethnic groups has also been documented (*8*). Strategies that prohibit the sale of flavored tobacco products, restrict price promotions, and implement culturally tailored antismoking campaigns can aid in reducing tobacco use disparities (*8*,*9*).

The findings in this report are subject to at least seven limitations. First, changes in weighting and design methodology for the 2019 NHIS could affect comparisons of weighted survey estimates over time; preliminary evaluation showed that the estimate of current cigarette smoking was affected by methodological changes, which might have shifted the estimate upward by 0.5 percentage points.^§§§§^ This small shift could account for the observed increase from 13.7% in 2018 to 14.2% in 2019. Second, changes in the 2020 NHIS survey administration from in-person to primarily telephone-based might affect estimates. Under- and overrepresentation of certain groups exists. How this might bias the measured prevalence of current cigarette smoking is uncertain^¶¶¶¶^ (*5*). For these reasons, observed trends should be interpreted cautiously. Third, there was a low response rate (29.6%) among adults reinterviewed from 2019 for the 2020 NHIS. Fourth, because NHIS is limited to the noninstitutionalized U.S. civilian population, results are not generalizable to institutionalized populations and persons in the military. Fifth, responses to questions were self-reported. However, research has shown that self-reported smoking status correlates highly with biochemical testing for serum cotinine (*10*). Sixth, multivariate analyses were not conducted. Finally, non-Hispanic adults categorized as of “other” races and non-Hispanic AI/AN adults have smaller sample sizes and lower statistical power for assessing differences. Related to this, the current definition of AI/AN excludes persons indicating both AI/AN and another race and ethnicity, further reducing the sample size and statistical power for the AI/AN group assessed in these data.

Continued monitoring of tobacco product use and tailored strategies and policies that reduce the effects of inequitable conditions (e.g., poverty, housing, and access to health care) could further aid in reducing disparities in tobacco use (*4*,*8*). Equitable implementation of comprehensive commercial tobacco control interventions, including smoke-free policies for public places and access to cessation services, is essential for maintaining progress toward reducing tobacco-related morbidity and mortality in the United States (*8*,*9*).

**Summary**

**What is already known about this topic?**

Although cigarette smoking has declined over the past several decades, a diverse landscape of combustible and noncombustible tobacco products has emerged in the United States.

**What is added by this report?**

In 2020, 19.0% of U.S. adults (47.1 million) used any tobacco product. Cigarettes were the most commonly used tobacco product (12.5%), followed by e-cigarettes (3.7%). From 2019 to 2020, the prevalence of overall tobacco product use, combustible tobacco product use, cigarettes, e-cigarettes, and use of two or more tobacco products decreased.

**What are the implications for public health practice?**

Continued monitoring of tobacco product use and tailored strategies and policies that reduce the effects of inequitable conditions could aid in reducing disparities in tobacco use.

## Footnotes

* The use of regular pipe, water pipe, or hookahs was assessed together using a single question. Interviewers could read the following sentences if necessary: “A hookah is a type of water pipe. It is sometimes called a ‘narghile’ (NAR-ge-lee) pipe. Do not include electronic hookahs or e-hookahs”; “Do not include electronic pipes or e-pipes”; “Do not include pipes filled with substances other than tobacco.”

† Categories are not mutually exclusive.

§ Current use of two or more tobacco products was defined as use either every day or some days of at least two or more of the following tobacco products: cigarettes (100 or more cigarettes during lifetime); cigars, cigarillos, or filtered little cigars; pipes, water pipes, or hookahs; e-cigarettes; or smokeless tobacco products.

¶ Hispanic persons could be of any race. All other groups were non-Hispanic. The following four non-Hispanic single-race categories were available for sample adults in the 2020 NHIS public use files: 1) White; 2) Black or African American; 3) Asian; and 4) AI/AN. Exclusive from these groups, the “non-Hispanic, Other” category includes those adults who were categorized as “non-Hispanic AI/AN and any other group” or “other single and multiple races.”

**
https://www.cdc.gov/nchs/nhis/data-questionnaires-documentation.htm

†† Because of the COVID-19 pandemic, data collection procedures in 2020 were disrupted, and from April through June all interviews were conducted by telephone only; from July through December, interviews were attempted by telephone first, with follow-ups to complete interviews by personal visit, when possible. During July–December, in-person data collection took place in selected areas based on COVID-19 rates; however, most interviews remained telephone-only. In addition, approximately one half of the original sample allocated for the last 5 months of 2020 was replaced with a reinterviewed sample (sample adults who completed the 2019 NHIS Sample Adult interview) because of concerns about possible loss of coverage and lower response rates typically associated with telephone interviewing. Additional details about NHIS 2020 interviewing during the COVID-19 pandemic are available at https://www.cdc.gov/nchs/nhis/data-questionnaires-documentation.htm.

§§ https://www.census.gov/programs-surveys/economic-census/guidance-geographies/levels.html; https://www2.census.gov/geo/pdfs/maps-data/maps/reference/us_regdiv.pdf

¶¶ Urban = large central metropolitan, large fringe metropolitan, medium metropolitan, and small metropolitan; rural = nonmetropolitan. Metropolitan statistical areas are based on the 2013 National Center for Health Statistics Urban-Rural Classification Scheme for Counties. https://www.cdc.gov/nchs/data/series/sr_02/sr02_166.pdf

*** Based on the imputed sample adult family income (grouped) variable (n = 31,568).

††† Sexual orientation was determined using the question, “Which of the following best represents how you think of yourself?” Response options included “gay,” “straight, that is, not gay,” “bisexual,” “something else,” and “I don’t know the answer” among male respondents, and “lesbian or gay,” “straight, that is, not lesbian or gay,” “bisexual, “something else,” and “I don’t know the answer” among female respondents. Respondents were considered to be lesbian, gay, or bisexual if they responded “gay,” “lesbian or gay,” or “bisexual.”

§§§ Private insurance coverage includes adults who had any comprehensive private insurance plan (including health maintenance organizations and preferred provider organizations). Medicaid for adults aged <65 years includes adults who do not have private coverage, but who have Medicaid or other state-sponsored health plans including Children’s Health Insurance Program; for adults aged ≥65 years, Medicaid includes adults aged ≥65 years who do not have any private coverage but have Medicare and Medicaid or other state-sponsored health plans. Medicare coverage only includes adults aged ≥65 years who only have Medicare coverage. Other public insurance includes adults who do not have private insurance, Medicaid, or other public coverage but have any type of military coverage, coverage from other government programs, or Medicare (adults aged <65 years). Uninsured includes adults who have not indicated that they are covered under private health insurance, Medicare, Medicaid, a state-sponsored health plan, other government programs, or military coverage. Insurance coverage is as of time of survey.

¶¶¶ Disability was defined based on self-reported presence of selected limitations including vision, hearing, mobility, remembering, self-care, and communication. These six questions are based on the short set of questions recommended by the Washington Group on Disability Statistics (https://www.cdc.gov/nchs/washington_group/index.htm). Further information on the coding is available at https://www.washingtongroup-disability.com/fileadmin/uploads/wg/Documents/WG_Resource_Document__4_-_Monitoring_Using_the_WG_Questions.pdf.

**** Regularly having feelings of anxiety and regularly having feelings of depression was assessed using questions from The Washington Group Short Set on Functioning – Enhanced: Question Specification for question source (https://www.washingtongroup-disability.com/fileadmin/uploads/wg/Documents/Questions/WG_Implementation_Document__4C_-_WG-SS_Enhanced_Question_Specifications.pdf). Further information on the definition is available at https://wwwn.cdc.gov/NHISDataQueryTool/ER_Quarterly/index_quarterly.html.

§§§§ 2019 NHIS documentation indicates that changes to the nonresponse adjustment approach and the calibration methods for the 2019 NHIS have the potential to affect comparisons of the weighted survey estimates over time. Preliminary evaluation showed that the estimate of current cigarette smoking was affected by the weighting adjustment. Estimates from 2019 might have shifted upward by 0.5 percentage points for the cigarette smoking prevalence estimate. This small shift because of methodological changes might account for the observed increase from 13.7% in 2018 to 14.2% in 2019. Similarly, the change in estimates for e-cigarette use from 3.2% in 2018 to 4.4% in 2019 might be due to methodological changes. Because of the changes in weighting and design methodology, direct comparisons between estimates for 2019 and earlier years should be made with caution because the impact of these changes has not been fully evaluated at this time. https://www.cdc.gov/nchs/data/nhis/earlyrelease/EReval202009-508.pdf; https://ftp.cdc.gov/pub/Health_Statistics/NCHS/Dataset_Documentation/NHIS/2019/srvydesc-508.pdf; https://www.cdc.gov/nchs/data/nhis/earlyrelease/EarlyRelease202009-508.pdf
